# Is Religiosity in a Prospective Partner Always Desirable? The Moderating Roles of Shared Social Identity and Medium of Communication when Choosing Interaction Partners

**DOI:** 10.1007/s12144-016-9437-z

**Published:** 2016-04-05

**Authors:** Chris Stiff

**Affiliations:** School of Psychology, Keele University, ST5 5BG, Keele, UK

**Keywords:** Religion, Group, Social identity, CMC

## Abstract

The profession of religion gives rise to myriad inferences and connotations, yet surprisingly little research has examined how it may influence with whom we choose to work. Two experiments conducted at a UK university investigated how religiosity by prospective collaborators affected attitudes and behaviour towards them. Participants in experiment 1 (*N* = 96) and experiment 2 (*N* = 120) demonstrated that individuals have a greater preference for, and are more likely to choose, a partner who shares their religious tendencies, but only when they anticipate working face-to-face. When electronic communication was anticipated, this bias disappeared. The implications for these findings are then discussed, particularly with regard to how they may impact on real-life issues such as online recruitment.

Religion is one of the most discussed– and most controversial – facets of modern life. In the 2011 UK Census, three-quarters of the population of the UK reported they followed some kind of religion, which included 33 million Christians, and 2 million Muslims (“Religion in England and Wales”, 2011). The ubiquity of religion is enormously influential (Lynch 2001), and clearly this prevalence warrants close attention from psychology.

Religion has been shown to affect both attitudes and behaviour. For example, in a meta-analysis Davis et al. (2013) demonstrated that religiosity correlated positively with forgiving others for past misdeeds, and also for forgiving oneself for transgressions. Being religious may also be an indicator of an individual’s propensity for pro-sociality and cooperation with others (see Galen 2012). Fitzgerald and Wickwire (2012) found that in a Trust Game, participants were more willing to trust, and would risk larger amounts of their endowments, when their partner in the games claimed to be religious.

Other studies have demonstrated the positive connotations associated with religiosity. In a study by Bailey (1985), an audience was presented with a target photograph, along with a description that indicated the target was either religious or not (depending on condition). Participants rated the religious target as more likeable, intelligent, trustworthy, and moral than non-religious targets. In a related study by Cook et al. (2000), college students were asked to brainstorm the ideas and concepts they immediately thought of when considering a hypothetical target who professed religiousness. These impressions were overwhelmingly positive, with participants giving greater weight to traits such as “peacefulness” and “striving for good”.

Religiosity does not always have positive connotations however. A study by Altermeyer and Hunsberger (1993) measured religiousness of participants and right-wing authoritarianism (comprising three components: the willingness to submit to a powerful leader, willingness to aggress to support that leader, and dislike of changes to the status quo), and found a positive correlation between the two. Laythe et al. (2001) measured levels of religious ardor in participants and prejudice towards other races, and likewise found that the former predicted the latter. A further study in a similar vein by Marsh and Brown (2011) investigated the links between strength of religious attitudes and discrimination towards homosexuals. Again, a significant positive predictive relationship was found. These links are well known outside of academic texts, often becoming apparent through the teaching religious followers subscribe to (e.g. the Catholic church’s stance regarding same-sex marriages, which is becoming increasingly contrary to its general approbation by Western society). Clearly, the relationship between an individual’s religiousness and their attractiveness as an interaction partner is a complex one.

## Choosing New Group Members

When encountering new people, we are adept at using any social information that is available to form an impression of that person (Sinha and Naykankuppam 2014). Impressions are formed extremely quickly when meeting others, with some research suggesting judgments can be made within 39 milliseconds of meeting a person (Bar et al. 2006). A variety of inferences can be made purely on an individual’s physical appearance (e.g. McConnell et al. 2008), or through reading just a few words about the target (Asch and Zukier 1984). Inference processes also appear to be automatic, and can occur even if explicit instructions are given *not* to form them (Crawford et al. 2007).

When choosing whom to work with, individuals may attempt to detect specific traits in their potential interaction partners (Zander 1976). Working with others offers fitness advantages (Buss 1999), but is also risky lest our partner exploit us for their own gain (Schnake 1991). So, rather than working indiscriminately with anyone, we appraise potential interaction partners for the indications that their behaviour will be aligned with our goals. For example, a person looking for a new business partner may attempt to detect financial acumen through the examination of candidates’ qualifications. By contrast, an individual seeking a person to live with may look for clues that indicate friendliness or sociability.

It has also been suggested that there are more general characteristics that are desirable regardless of the type of interaction. A strong moral code that indicates an individual will “do the right thing” is almost always seen as a positive trait (Leach et al. 2007). Similarly, a strong sense of commitment towards a partnership is also a universally desirable quality (Nesse 2001). Yamagishi and Hayashi (1996) examined a variety of factors at play when choosing whom to work with on mixed-motive experimental games, and found the most attractive player was one who was “nice, prudent, and trustful” (pg. 378). That is, the most desirable individuals are ones who are cooperative with others, impart trust to potential partners, but are also capable of retaliating when betrayed.

## The Desirability of Religiousness – Social Identity Effects

Clearly, humans are capable of making rapid judgments about others; they are also capable of utilizing whatever information is available to make those judgments however sparse it may be. Given this predisposition, and given the salience of religion in society, it seems highly likely we may make judgments about whom to interact with based on their religion if that information is available. Surprisingly, there is little in the literature that has investigated this idea. Most existing work has examined how religion may impact on relations between pre-existing, static groups. The aim of this work is to address this issue, and examine religion in a *dynamic* context. How might it influence whom we choose to enter into collaborative endeavors with?

Several lines of work have indicated that religion can be conceptualized as a *social identity*, and accordingly displays the same biases and behavioral influences as other, secular identities. Ysseldyk et al. (2010) commented that religion offers clear markers and boundaries of membership, has prescribed normative behaviours, and enhances the self-concept and self-esteem of those that adhere to it, much as other social identities do. More specifically, effects like *in-group bias* are often present in religions. According to Social Identity Theory (Tajfel 1978; Tajfel and Turner 1979), individuals are motivated to enhance the perception of groups with which they identify. This may manifest itself in preferential treatment for other in-group members, and poorer treatment for those in the out-group (see also Brewer 1979; Scheepers et al. 2006).

This idea has been born out empirically. Fitzgerald and Wickwire (2012) asked participants to play a Trust Game with a partner who shared their particular faith (in this case, Baptism) or did not. They found individuals donated more to their partners, and reciprocated trust to a higher degree when religion was shared compared with when they played with a non-religious partner. Moreover, the in-group bias displayed was greater than when the shared identity was trivial, such as in a minimal group paradigm. Widman et al. (2009) asked individuals who scored highly on the Doctrinal Orthodoxy scale (which measures strength of religiousness; Batson et al. 1993) to rate a target who prominently displayed a crucifix pendant in their photo. Such targets were rated greater for kindness and morality than similar targets who did not display a crucifix. Moreover, this effect was only present for high scorers on the Doctrinal Orthodoxy Scale; low scorers showed no such effect. Thus in both these studies, individuals who showed strong allegiance to their religion displayed in-group bias, akin to that shown with other social identities.

If religion was seen as a universally positive (or negative) characteristic, then a potential interaction partner who professed religion would always be seen as more (or less) desirable than a non-religious alternative, and would be more likely to be chosen to work with. However, we hypothesize that the religiousness of the individual choosing will also be influential. That is, an in-group bias is likely to be displayed. Highly religious individuals will rate religious candidates as the most desirable, whereas less religious individuals will most likely prefer non-religious candidates (Hypothesis 1)

## Other Influences on Choosing an Interaction Partner

As well as the religiousness of a potential partner and the religiousness of the individual choosing a partner, there is a third factor which we wish to explore in this work: medium of communication. Increasingly, individuals working on collaborative tasks may not interact directly, but instead may communicate over the Internet using email, or “chat” programs. The effect of this on individuals’ behaviour has sparked a wealth of psychological research, particularly in comparing the performance of geographically dispersed collaborators with their face-to-face (F2F) equivalents.

The research falls into two camps. Some researchers suggest that individuals who communicate via computer-mediated-communication (CMC) will share weaker bonds, and be less cohesive that individuals who work together F2F due to the reduction in non-verbal cues available via that media – the *cue filtered out* perspective (e.g. Sproull and Kiesler 1986). This may then lead to larger degrees of non-cooperation and social loafing compared with individuals working in close proximity (e.g. Alnuaimi et al. 2010; Naquin et al. 2008; Suleiman and Watson 2008). However, other researchers have suggested that CMC partnerships may be *as* effective – or even *more* effective - than F2F teams. The Social Identity Model of Deindividuation Effects (SIDE model; Lea et al. 2001) argues that in the presence of anonymity, a salient social identity can increase conformity to group norms. Individuals in such conditions may therefore act in a more pro-social, cooperative fashion with other group members (see Chan 2010 for an example).

If CMC does tend to weaken social bonds as per the cues-filtered-out perspective, then we can anticipate that any effects found for religion of a candidate and/or the participant will be smaller when participants are told they will be working with their partner via email (Hypothesis 2a). By contrast, if CMC enhances the effects of salient social information as per the SIDE model, then any effects should be *greater* compared with a F2F condition (Hypothesis 2b).

## Summary and Overview of the Current Work

In this paper, we aim to examine a novel aspect of religion in social psychology; namely, its influence in the dynamic context of *choosing* interaction partners. How will a participant respond to a potential interaction partner who professes to be religious, compared with one who does not? We will also contribute to our understanding of how communication medium may moderate these effects. Will telling participants they will be working with their partner via email enhance or dilute the influence of religion on their decisions?

To examine these ideas, participants were asked to make judgments about potential partners who were said to be religious, or not. The method by which they would be communicating with these partners was also manipulated; either they would be working face-to-face, or via email. We expected that the religion of the candidate, the religious leanings of the participants, and the medium of communication would all have significant influences on these decisions.

## Experiment 1

### Method

#### Participants

One hundred and twenty-six participants initially entered into this experiment. Participants were divided equally between the religious and non-religious condition depending on their responses regarding their own religiousness (see below). Eleven participants failed to return their screening survey in time and were removed from the experiment. A sufficient number of people were found for the non-religious condition first; any participants after this who were classified as “non-religious” (of which there were 19) were channeled into an entirely separate study when they arrived at the lab for their session, and did not take part in this procedure further. In total, 96 participants took part in the complete experiment (28 male), with an age range of 18 to 55 years (*M* = *20.84*, *SD* = 5.65, *Median* = *19 years*). The religion of participants was not specifically recorded, but it was assumed the majority of religious participants were Christian due to the typical distribution of religions in the UK. All participants were students at a north west UK university, and took part in return for either course credit, or for a payment of £5 ($7.82).

#### Design

This experiment used a 2 (participant religious vs. non-religious) × 2 (proposed partner religious vs. non-religious) × 2 (face to face vs. computer-mediated communication) fully independent design. The main dependent variables were participants’ responses to the items “how much would you like to work with this person?” and “how desirable do you find this person as a partner?”.

#### Materials

Participants were provided with a short survey about themselves before taking part in the main study; this contained a number of filler questions, along with the 10-item Religious Faith Questionnaire which measures the strength of a respondents’ religious faith (Plante and Boccaccini 1997a, b, see Appendix), distributed throughout the survey (e.g. “I pray daily”). Participants indicated to what extent they agreed or disagreed with these items on 4-point rating scale (1 = strongly disagree, 2 = disagree, 3 = agree, 4 = strongly agree). Participants’ scores were summed with the maximum score being 40. Instructions for the scale suggest low and high religiousness are given by absolute scores on the scale. Therefore, participants scoring below the midpoint (i.e. below 20) were classified as non-religious. Those that scored above the midpoint (i.e. scored 20 or more) were classified as religious. Information about the experiment, the participants’ proposed partners, and all response sheets were provided on paper.

#### Procedure

Informed consent was obtained from all individual participants included in the experiment. Two days before taking part, participants were emailed and asked to promptly return a short survey which contained the Religious Faith Questionnaire hidden amongst other filler items.

On the day, participants arrived and were seated in individual cubicles.

All instructions were relayed face-to-face by the experimenter, and participants were told they could ask for clarification on any aspect at any time. Participants were (falsely) told there were three other people taking part in their session, which was looking at “how we make judgments about other people”. They would be supplied with information about these other three people, and asked to rate how desirable they thought they would be to work with based on this information. The participants were told they would also *provide* similar information about themselves, and similarly be rated. The experimenter would then randomly pair individuals, and they would work together on a “collaborative task”. Details on this task were left deliberately vague, but participants were told it would require “working together” and if they were successful at it they would receive a £10 bonus.

After being told about the structure of the experiment, participants were allocated to one of the four conditions in an orthogonal fashion. Participants were first either told they would be working in the same room as their partner on the subsequent collaborative task (face-to-face condition) or they would be communicating electronically and would *not* meet (email condition). It was stressed that the partner would *not* be told anything about the ratings the participant had given them at any point. Participants were then supplied with a piece of paper headed “what is your dream job?” and asked to write an answer of no more than five lines. When finished, this was taken out of the cubicle and participants were supplied with information ostensibly from one of the other individuals taking part. The paper was headed “what is a hobby or pastime you enjoy?” with the following answer. In the non-religious condition, the words in parentheses were absent. As the data was gathered from a UK city, and would be generalised to the population of that country, we used attributes that tended to occur in that sample/population to avoid effects that might occur had we used other religions that may be synonymous with ethnicity/minorities:

“I am a member of a (Bible) reading group which meets once a week. I think (Bible) reading is very important and enjoyable, and something that is an important part of me. I read (the Bible) quite a lot outside of the group, and sometimes discuss what I’ve read with other people”

Participants were then asked to respond to two items regarding their partner: “how much would you like to work with this person?” and “how desirable do you find this person as a partner”?, both responded to on a 5-point rating scale (1 = not at all, 2 = very little, 3 = somewhat, 4 = a lot, 5 = very much so). Once they had completed this, they were informed that was actually the end of the experiment, and no others were taking part. They were then debriefed, paid and dismissed.

### Results

An initial analysis including gender showed no significant main effects or interactions for this variable (all F’s < 1). Therefore, it was excluded from the subsequent section. A chi-square analysis examining differences in gender between religious and non-religious conditions was non-significant (χ^2^ (1, *N* = 96) = .20, ns).

Participants’ responses to the item “how much would you like to work with this person?” and “how desirable do you find this persona as a partner” were highly correlated (*R* = .63, *N* = 96, *p* < .01) and so were averaged, to create a single “desirability” index. These values were entered into a 2 (participant religious vs. non-religious) × 2 (proposed partner religious vs. non-religious) × 2 (face to face vs. computer-mediated communication) between subjects ANOVA.

This yielded a significant main effect of communication medium (*F* (1, 88) = 5.03, *p* = .03, *η2* = .05). Participants rated the potential partner more positively if they were anticipating working face to face (*M* = 3.98, *SD* = .95) compared with via email (*M* = 3.54, *SD* = .94). There was also a significant partner’s religiousness x participant’s religiousness interaction (*F* (1, 88) = 10.95, *p* < .001, *η2* = .11), which was then subsumed by a significant three-way interaction (*F* (1, 88) = 4.11, *p* = .04, *η2* = .05). All other effects and interactions were non-significant.

To examine this more closely, an ANOVA was performed on each communication medium condition separately. For the face-to-face condition, this provided a significant partner’s religiousness x participant’s religiousness interaction (*F* (1, 44) = 14.02, *p* < .001, *η2* = .24). When the participant was religious, they gave significantly higher ratings for a religious partner compared with a non-religious partner. When the participant was not religious, the opposite was true - see Table 1.

**Table 1:** Means and standard deviations for participant’s religiousness and partner’s religious when face-to-face communication was anticipated

		Partner’s religiousness		t
		Religious	Non-religious	
Participant’s religiousness	Religious	4.67 (.89)	3.58 (1.08)	2.68*
	Non-religious	3.33 (1.15)	4.33 (.65)	2.63*
t		3.71**	2.06	

Figures refer to participants’ responses to the item “How much would you like to work with this person”, given on a Likert scale from 1 to 6 with a higher score indicating a more positive response. Figures in parentheses are SD

* = *p* < .05. ** = *p* < .01

For the email condition, there were no significant main effects nor interactions. (all *F*’*s* < 1.5, see Table 2).

**Table 2:** Means and standard deviations for participant’s religiousness and partner’s religious when email communication was anticipated

		Partner’s religiousness		t
		Religious	Non-religious	
Participant’s religiousness	Religious	3.58 (.79)	3.67 (.98)	.23
	Non-religious	3.17 (.83)	3.75 (1.14)	1.43
t		1.25	0.19	

Figures refer to participants’ responses to the item “How much would you like to work with this person”, given on a Likert scale from 1 to 6 with a higher score indicating a more positive response. Figures in parentheses are SD

* = *p* < .05. ** = *p* < .01

### Discussion

The results from experiment 1 supported hypothesis 1. Participants overall demonstrated a preference for partners who shared their religious beliefs (or lack of such beliefs). This effect was then further moderated by the medium of communication used. Participants demonstrated an in-group bias when told they would be working face-to-face with their partner in the future. However, when participants believed they would be communicating with their partner electronically, social identity effects were significantly attenuated. This supports a *cues-filtered-out* perspective for this situation; hypothesis 2a.

Experiment 2 aimed to shed more light on these findings using some methodological adjustments. First, we used a different statement to indicate the supposed partner’s religiousness, to enhance the generalizability of the findings. Second, we were concerned that participants in experiment 1 were not making a direct decision about who to work with; rather, they were indicating a preference. To remedy this, in experiment 2 participants were given a *choice* between a religious and non-religious partner when deciding who to work with, rather than simply responding on an attitudinal measure.

The hypotheses for experiment 2 were the same as the previous experiment; namely, that participants would demonstrate a preference for others who shared their religious ideologies when told they would be working face-to-face (hypothesis 1), but that this effect would be attenuated when told they would be working electronically (now simply termed hypothesis 2).

## Experiment 2

### Method

#### Participants

One hundred and seventy-four participants signed up for experiment 2. As with the previous experiment, participants were initially supplied with a short survey interspersed with items from the Religious Faith Questionnaire (Plante and Boccaccini 1997a, b) and then funneled into the appropriate condition. Fourteen failed to return their surveys in time. The non-religious condition was again filled first; 16 subsequent “non-religious” participants were then assigned to an entirely different study upon arriving in the lab.

This left 144 participants who actually took part (89 female). Participants were students attending a north west university in the United Kingdom and took part in return for course credit, or £5 ($7.82). Participants mean age was 24.45 years, varying between 18 and 42 years (*SD* = 3.22, *Median* = *20*).

#### Materials

The materials from experiment 1 were used here, with participants being provided information and response sheets on paper.

#### Design

The main variables in this experiment were the participants’ religiousness and the medium of communication they would be using. Unlike experiment 1, participants were presented with a religious *and* non-religious candidate to work with, and asked to choose between the two. Thus, experiment 2 utilized a 2 (participant religious vs. non religious) × 2 (communicating face to face vs. by email) × 2 (candidate religious vs. non religious) mixed design. The main dependent variable was the choice participants made between candidates.

#### Procedure

Informed consent was obtained from all individual participants included in the experiment. Experiment 2 initially followed experiment 1 in that participants were asked about their own religious preferences by responding the Religious Faith Questionnaire items (hidden amongst other filler items) two days prior to taking part in the main experiment, and upon arriving were told they would be shortly working with a partner on a collaborative endeavor. The medium of communication manipulation was introduced in a similar fashion; participants were told that the subsequent task would involve working with their partner either face-to-face, or using email.

However, after this the method differed. Participants were told (falsely) that they were in a session with two other people. One of the three of them would be designated the “chooser” and would pick a partner from the other two for the collaborative task. The remaining individual would then be assigned to some other task to work alone. Participants picked a piece of paper from a hat that designated them the chooser (in reality, this was fixed so all pieces of paper gave this role to the participant). To help them choose, participants were told they would be given some information about each potential partner and asked to pick one.

Participants were then presented with two pieces of paper, ostensibly written by the potential partners (different handwriting was used to reinforce this idea) detailing a hobby or interest they enjoyed. The religious partner wrote the following:

“I spend a lot of time with my church group. We meet after church every week, and there’s usually an organised outing once a month”.

The non-religious partner wrote one of the following, (chosen by the experimenter at random from a box of folded up slips of paper:

“I enjoy walking and seeing the countryside. I sometimes go alone or with friends”

“Most weekends I see a film with friends, then we have a meal afterwards”

“My friends and I play soccer once a week after work, in the evening”.

Participants then chose which individual they would like to work with on the collaborative task. Once participants had confirmed their choice, they were told that in fact there was no further task, and the experiment was over. They were then fully debriefed as to the true nature of the experiment, compensated, and dismissed.

### Results

The number of times a religious partner was chosen over a non-religious one was tallied, and then fed into a three-way loglinear analysis. This produced a final model indicating a three-way (highest-order) interaction between participants’ religiousness, medium of communication, and their choice of a religious partner (*χ2* (1, *N* = 144) = 3.60, *p* = .04). To break this effect down, two separate chi-square analyses were performed for each medium of communication.

In the face-to-face condition, the chi-square value was significant (*χ2* (1, *N* = 72) = 10.92, *p* = .001). Participants were more likely to choose a religious partner if they themselves were religious, and were more likely to choose a non-religious partner if they were not – see Table 3.

**Table 3:** Participants’ own religion and subsequent choices between a religious and non-religious partner (face-to-face condition only)

		Number of times partner type was chosen	
		Religious	Non-religious
Participant’s religion	Religious	26	10
	Non-religious	12	24

The same chi-square was performed on the choices for participants in the electronic communication condition. Although the preferences of participants showed a similar pattern as the face-to-face condition, the chi-square statistic was not significant (*χ* 2 (1, *N* = 72) = .50, *ns*). –see Table 4.

**Table 4:** Participants’ own religion and subsequent choices between a religious and non-religious partner (email condition only)

		Number of times partner type was chosen	
		Religious	Non-religious
Participant’s religion	Religious	20	16
	Non-religious	17	19

### Discussion

In experiment 2, participants were asked to directly *choose* which individual they wished to work with, rather than simply indicate a preference as they had in the previous study. This offered a more rigorous measure of their desire to work with another person based on their (and their proposed partner’s) religiousness, and how this might interact with the medium of communication.

Our main hypotheses were again supported. Participants showed a greater preference for an individual sharing their religious ideology, providing further evidence of social identity effects for religion (hypothesis 1). The medium of communication again moderated this effect (hypothesis 2); when participants were told they would be working with their partner electronically, in-group bias essentially vanished. Thus it appears in this context that social identity effects are considerably diluted when participants anticipate indirect communication with their partner-to-be.

## General Discussion

The influence of religion in social psychology is something that has spawned a good deal of attention; however very little research has examined how it might influence choosing a partner to work with. The literature had tended to view intra- and inter-group relations in a static context, wherein membership does not change. This work aimed to address this by studying the influence of religiousness in the dynamic formation of collaborative dyads, whereby individuals make choices regarding whom they interact with. We aimed to examine how the medium of communication may moderate this influence.

Two studies showed strong support for the hypothesis that religion *is* highly influential when choosing interaction partners, but is also dependent on the religion of the individual choosing, and on their anticipated form of communication. When participants were told they would be working face-to-face with their partner, they showed a marked preference for an individual sharing their own religious ideology. However, when told they would be communicating electronically, this preference disappeared. These effects were demonstrated both when participants rated potential partners (experiment 1) and when they explicitly chose who they wanted to work with (experiment 2).

### Integrating Findings with Previous Literature

At first blush, the findings here may seem to contradict the SIDE model of suggested by Lea et al. (2001). Here, social identity effects are seemingly *enhanced* when individuals communicate electronically, as the anonymity afforded by the Internet increases depersonalization and heightens the salience of a social identity. However, there are a number of elements in the current work which account for this.

The SIDE model highlights the importance of anonymity and depersonalization in its mechanisms (Robertson 2006). In the current work, participants were not anonymous – the experimenters were aware of their identity, and they themselves were told they were playing an important role in rating/choosing their partners. Matheson and Zanna (1990) have suggested this would decrease depersonalization and heighten self-awareness, so participants would be making decision on a personal rather than social level. The SIDE model is also reliant on groups espousing a strong and consistent norm; anonymity then heightens the conformity of group members to this norm. No such norm was presented in this work; participants were simply told they would be working with another individual, and asked to make decisions regarding that eventuality.

Thus it appears likely then when individuals work in a large collective online, where depersonalization and anonymity are heightened, the SIDE model can be applied. By contrast, when individuals are self-aware, identifiable, but communicating electronically, social identity effects are greatly reduced. This is consistent with other work which has examined electronic communication amongst identifiable participants. For example Bhappu et al. (1997) found that when discussing controversial topics, participants’ social identities (in this case, their gender identity) moderated their attention and responses when face to face, but that this effect was absent when communicating electronically.

### Sub- and Super-ordinate Identities in Religion

In this work, the expression of religiousness from the supposed partner had considerable Christian overtones; the participants by contrast were only asked if they were religious or not, rather than their specific denomination or ideology. What effect may this have had?

The Common Ingroup Identity Model argues that the adoption of a superordinate identity can increase pro-social tendencies amongst those that share it. Therefore, the identity of “religious” would lead to in-group bias, regardless of the *type* of religion involved. Given the effects found here, it seems likely that this process occurred amongst participants. However, biases can exist between sub-groups, as much as they can between ingroups and outgroups. Huddy and Virtanen (1995) demonstrated that Latino sub-groups show just as much bias towards their own sub-group (compared with other sub-groups) as White Americans do to Latinos as a whole. Rabinovich and Morton (2011) have also argued that when superordinate identities are too diffuse to be meaningful (for example, on the national level) this may not be an effective method of eliciting pro-sociality, and sub-ordinate identity priming may be more suitable. Therefore, if the specific religion being professed by the potential partner(s) was made more salient, we may find in-group bias at the sub-group level, with non-religious partners treated in an equivalent manner to other religious individuals outside the participant’s sub-group.

#### Methodological Issues and Future Work

Although the current work has made a unique contribution to our understanding of how religion affects social preferences, and how medium of communication may moderate these preferences, there are nevertheless improvements that could be made to its methodology. In these experiments, participants did not actually engage with real partners; the information provided was contrived by the experimenter. It may be worthwhile obtaining genuine information from participants regarding their outside interests and examining whether the religiousness expressed in these influence choices in a manner comparable to our own manipulations.

The sample chosen for data collection may also be important. The majority of the participants were relatively young, and attending university. Research suggestions that older adults may be more influenced by religious tendencies and have religion feature as part of their lives to a greater extent (Schlehofer et al. 2008). Students may also tend to be less religious than those who do not attend university (Frankel and Hewitt 1994). Therefore we may wish to consider these influences in any future work on this topic.

A second extension may be to look how participants actually work together. In this work, no collaborative task was undertaken; the experiment stopped once participants had given their responses. It would be interesting therefore to examine how participants might behave towards the partner they had chosen. In the face-to-face conditions, participants appeared more favourably disposed to partners who shared their religious tendencies; presumably they would therefore behave in a more favorable manner also. However, it is possible participants would presume their partner would act benevolently, and actually *exploit* them because of this (Utz et al. 2004). That is, if participants are expecting their partner to work hard on the collaborative task (because of their shared identity), they may deliberately *under*perform to maximize their own gains with minimal expenditure of their own resources; they may in fact *socially loaf* (Karau and Williams 1995). Of course, acting in this manner is contrary to normative expectations, but that does not mean it would not occur, particularly if the participants did not strongly identify with the group (Widman et al. 2009). Further studies could investigate this possibility in more detail.

In this paper we have examined dyads; however *groups* of individuals frequently acquire new members (Levine and Moreland 1994), and the context of this recruitment may moderate the use of religiosity information. Zander (1976) has remarked that groups whose performance is measure in objective terms (e.g. the number of units sold by a sales team) tend to appraise potential new members in objective terms too. In such groups, it may be that the religiosity of newcomers is reduced in salience. Similarly, Cini et al. (1993) have suggested that *understaffed* groups may be more receptive to members. In this case, it may be that a group that is struggling to perform will pay less attention to any religiosity mis-match in potential group members.

We can also consider implications for the increasing use of email amongst collaborating individuals in organisations. Although the “religiosity” cue seems to have been attenuated in our experiments, other cues – such as sex and status – do moderate responses to electronic communication in some cases (O’Neill and Colley 2006). Further examination of precisely what extra-message information is utilized when communicating in electronically would give us a useful insight into working practices in the 21st century.

### Conclusion

In this work, we have made a significant contribution to the literature by demonstrating the role of religiousness in *choosing* interaction partners, a thus far under-studied area in psychology. We have demonstrated that religiousness is *not* seen as a universally desirable trait akin to commitment or honesty, but is actually dependent on the religiousness of the perceiver. However, this effect only holds when anticipating face-to-face interactions; when an individual believes they will be working with a partner electronically, bias effects disappear. Future studies may wish to examine how these preferences and decisions may manifest should a subsequent collaborative task actually take place to further illuminate the role of religiousness in relationship dynamics.

## Appendix

### Religious Faith Questionnaire

Please answer the following questions about religious faith using the scale below. Indicate the level of agreement (or disagreement) for each statement.

## 1 = strongly disagree 2 = disagree 3 = agree 4 = strongly agree

1. My religious faith is extremely important to me.
2. I pray daily.
3. I look to my faith as a source of inspiration.
4. I look to my faith as providing meaning and purpose in my life.
5. I consider myself active in my faith or church.
6. My faith is an important part of who I am as a person.
7. My relationship with God is extremely important to me.
8. I enjoy being around others who share my faith.
9. I look to my faith as a source of comfort.
10. My faith impacts many of my decisions.

To score, add the total scores. They will range from 10 (low faith) to 40 (high faith).

## References

[CR1] Alnuaimi OA, Robert LP, Maruping LM (2010). Team Size, Dispersion, and Social Loafing in Technology-Supported Teams: A Perspective on the Theory of Moral Disengagement. Journal of Management Information Systems.

[CR2] Altermeyer B, Hunsberger B (1993). Reply to Gorsuch. The International Journal for the Psychology of Religion.

[CR3] Asch SE, Zukier H (1984). Thinking about persons. Journal of Personality and Social Psychology.

[CR4] Bailey R (1985). Perceptions of professionals who express religious beliefs. Social Behavior and Personality: An International Journal.

[CR5] Bar M, Neta M, Linz H (2006). Very first impressions. Emotion.

[CR6] Batson CD, Schoenrade P, Larry W (1993). *Religion and the individual*: *A social-psychological perspective* (Vol. viii).

[CR7] Bhappu AD, Griffith TL, Northcraft GB (1997). Media Effects and Communication Bias in Diverse Groups. Organizational Behavior and Human Decision Processes.

[CR8] Brewer MB (1979). In-group bias in the minimal intergroup situation: A cognitive-motivational analysis. Psychological Bulletin.

[CR9] Buss DM (1999). *Evolutionary psychology*: *The new science of the mind* (Vol. xxii).

[CR10] Chan M (2010). The impact of email on collective action: a field application of the SIDE model. New Media & Society.

[CR11] Cini MA, Moreland RL, Levine JM (1993). Group staffing levels and responses to prospective and new group members. Journal of Personality and Social Psychology.

[CR12] Cook SW, Borman PD, Moore MA, Kunkel MA (2000). College students’ perceptions of religious people and spiritual people. Journal of Psychology and Theology.

[CR13] Crawford MT, Skowronski JJ, Stiff C (2007). Limiting the spread of spontaneous trait transference. Journal of Experimental Social Psychology.

[CR14] Davis DE, Worthington EL, Hook JN, Hill PC (2013). Research on religion/spirituality and forgiveness: A meta-analytic review. Psychology of Religion and Spirituality.

[CR15] Fitzgerald CJ, Wickwire JH (2012). Religion and political affiliation’s influence on trust and reciprocity among strangers. Journal of Social, Evolutionary, and Cultural Psychology.

[CR16] Frankel BG, Hewitt WE (1994). Religion and well-being among Canadian university students: The role of faith groups on campus. Journal for the Scientific Study of Religion.

[CR17] Galen LW (2012). Does religious belief promote prosociality? A critical examination. Psychological Bulletin.

[CR18] Huddy L, Virtanen S (1995). Subgroup differentiation and subgroup bias among Latinos as a function of familiarity and positive distinctiveness. Journal of Personality and Social Psychology.

[CR19] Karau SJ, Williams KD (1995). Social loafing: Research findings, implications, and future directions. Current Directions in Psychological Science.

[CR20] Laythe B, Finkel D, Kirkpatrick LA (2001). Predicting Prejudice from Religious Fundamentalism and Right-Wing Authoritarianism: A Multiple-Regression Approach. Journal for the Scientific Study of Religion.

[CR21] Lea M, Spears R, de Groot D (2001). Knowing Me, Knowing You: Anonymity Effects on Social Identity Processes within Groups. Personality and Social Psychology Bulletin.

[CR22] Leach CW, Ellemers N, Barreto M (2007). Group virtue: The importance of morality (vs. competence and sociability) in the positive evaluation of in-groups. Journal of Personality and Social Psychology.

[CR23] Levine JM, Moreland RL (1994). Group Socialization: Theory and Research. European Review of Social Psychology.

[CR24] Lynch MJ (2001). Religion’s influence on culture and psychology. American Psychologist.

[CR25] Marsh T, Brown J (2011). Homonegativity and its Relationship to Religiosity, Nationalism and Attachment Style. Journal of Religion and Health.

[CR26] Matheson K, Zanna MP (1990). Computer-mediated communications: The focus is on me. Social Science Computer Review.

[CR27] McConnell AR, Rydell RJ, Strain LM, Mackie DM (2008). Forming implicit and explicit attitudes toward individuals: Social group association cues. Journal of Personality and Social Psychology.

[CR28] Naquin CE, Kurtzberg TR, Belkin LY (2008). E-Mail Communication and Group Cooperation in Mixed Motive Contexts. Social Justice Research.

[CR29] Nesse RM (2001). Evolution and the Capacity for Commitment.

[CR30] O’Neill R, Colley A (2006). Gender and status effects in student e-mails to staff: Gender and status effects in student e-mails to staff. Journal of Computer Assisted Learning.

[CR31] Plante, T. G., & Boccaccini, M. T. (1997a). The Santa Clara strength of religious faith questionnaire. *Pastoral Psychology, 45*(5), 375–387. 10.1007/s11089-021-00972-3 PMC8406649 34483371

[CR32] Plante, T. G., & Boccaccini, M. (1997b). Reliability and validity of the Santa Clara strength of religious faith questionnaire. *Pastoral Psychology, 45*(6), 429–437.

[CR33] Rabinovich A, Morton TA (2011). Subgroup identities as a key to cooperation within large social groups: Identity salience and shared resources. British Journal of Social Psychology.

[CR34] Robertson T (2006). Dissonance effects as conformity to consistency norms: The effect of anonymity and identity salience. British Journal of Social Psychology.

[CR35] Scheepers D, Spears R, Doosje B, Manstead AS (2006). Diversity in in-group bias: Structural factors, situational features, and social functions. Journal of Personality and Social Psychology.

[CR36] Schlehofer MM, Omoto AM, Adelman JR (2008). How do “religion” and “spirituality” differ? Lay definitions among older adults. Journal for the Scientific Study of Religion.

[CR37] Schnake ME (1991). Equity in Effort: The “Sucker Effect” in Co-Acting Groups. Journal of Management.

[CR38] Sinha J, Naykankuppam D (2014). The canny social judge: Predicting others’ attitudes from sparse information. Journal of Experimental Social Psychology.

[CR39] Sproull L, Kiesler S (1986). Reducing Social Context Cues: Electronic Mail in Organizational Communication. Management Science.

[CR40] Suleiman J, Watson RT (2008). Social Loafing in Technology-Supported Teams. Computer Supported Cooperative Work: The Journal of Collaborative Computing.

[CR41] Tajfel H (1978). Differentiation between social groups: Studies in the social psychology of intergroup relations (Vol. xv)..

[CR42] Tajfel H, Turner J, Austin W, Worchel S (1979). An integrative theory of intergroup conflic. The social psychology of intergroup relations.

[CR43] Utz S, Ouwerkerk JW, Van Lange PAM (2004). What is smart in a social dilemma? Differential effects of priming competence on cooperation. European Journal of Social Psychology.

[CR44] Widman DR, Corcoran KE, Nagy RE (2009). Belonging to the same religion enhances the opinion of others’ kindness and morality. Journal of Social, Evolutionary, and Cultural Psychology.

[CR45] Yamagishi, T., & Hayashi, N. (1996). Selective play: Social embeddedness of social dilemmas. In *Frontiers in social dilemmas research* (pp. 363–384). Springer. Retrieved from http://link.springer.com/chapter/10.1007/978-3-642-85261-9_19

[CR46] Ysseldyk R, Matheson K, Anisman H (2010). Religiosity as identity: toward an understanding of religion from a social identity perspective. Personality and Social Psychology Review.

[CR47] Zander A (1976). The Psychology of Removing Group Members and Recruiting New Ones. Human Relations.

